# *Shigella* spp. with Reduced Azithromycin Susceptibility, Quebec, Canada, 2012–2013

**DOI:** 10.3201/eid2005.130966

**Published:** 2014-05

**Authors:** Christiane Gaudreau, Sapha Barkati, Jean-Michel Leduc, Pierre A. Pilon, Julie Favreau, Sadjia Bekal

**Affiliations:** Centre Hospitalier de l’Université de Montréal–Hôpital Saint-Luc, Montreal, Quebec**,** Canada (C. Gaudreau, S. Barkati, J.-M. Leduc, J. Favreau);; Université de Montréal, Montreal (C. Gaudreau, S. Barkati, J.-M. Leduc, P.A. Pilon, S. Bekal);; Agence de la Santé et des Services Sociaux de Montréal-Santé Publique, Montreal (P.A. Pilon);; Laboratoire de Santé Publique du Québec/Institut National de Santé Publique du Québec, Sainte-Anne-de-Bellevue, Quebec, Canada (C. Gaudreau, S. Bekal)

**Keywords:** *Shigella* spp., emergence, azithromycin, drug resistance, men, Quebec, Canada, homosexual, MSM, bacteria

## Abstract

During 2012–2013 in Montreal, Canada, 4 locally acquired *Shigella* spp. pulse types with the *mph*(A) gene and reduced susceptibility to azithromycin were identified from 9 men who have sex with men, 7 of whom were HIV infected. Counseling about prevention of enteric sexually transmitted infections might help slow transmission of these organisms.

*Shigella* spp. are transmitted directly from person to person or indirectly by low-inoculum infection (*1*). Among men who have sex with men (MSM), *Shigella* spp. are mostly transmitted sexually; clusters of such cases have been documented in Montreal and surrounding neighborhoods (*2*,*3*). Azithromycin is an alternative treatment for multidrug-resistant *Shigella* spp. infections in adults and children, but routine testing for azithromycin susceptibility is not yet standardized and recommended (*1*,*4*–*6*). In the United States, azithromycin MICs for 392 wild-type *Shigella* strains isolated in 2005–2006 were estimated to be 4–16 mg/L; the azithromycin MIC for 90% of the isolates was 8 mg/L (*7*).

## The Study

In December 2012, the microbiology laboratory of the Centre Hospitalier de l’Université de Montréal–Hôpital Saint-Luc identified *Shigella* spp. with reduced susceptibility to azithromycin from 2 patients who had received this agent as treatment for shigellosis. The Montréal Public Health Department and Laboratoire de Santé Publique du Québec (LSPQ) were alerted. Retrospective and prospective laboratory surveillance was initiated to cover the period January 2011–April 2013. Laboratories routinely report shigellosis to the Montreal Public Health Department (Quebec, Canada).

Phenotypic identification of all *Shigella* spp. at the genus and species levels (*8*) was confirmed at LSPQ as described (*9*), after which serologic identification by slide agglutination (Denka Seiken Co., Ltd, Coventry, UK) was performed. Pulsed-field gel electrophoresis (PFGE) was performed at LSPQ according to international standards set by the US Centers for Disease Control and Prevention (*10*). Pulse types were determined by *Shigella* species, serotypes, and PFGE patterns. All *Shigella* spp. isolated during 2011–2013 underwent susceptibility testing for ampicillin, trimethoprim/sulfamethoxazole, and ceftriaxone by use of Vitek 2 (bioMérieux, Marcy l’Étoile, France) and for azithromycin and ciprofloxacin by use of Etest (AB Biodisk, Solna, Sweden). *Shigella* spp. with elevated MICs for azithromycin were also tested by disk diffusion for 30 μg nalidixic acid and by Etest for tetracycline and chloramphenicol. Vitek 2 and Etest susceptibility testing was performed as recommended by the manufacturers, and quality control strains gave expected results. The *mph*(A) gene, which codes for the macrolide 2′-phosphotransferase, was detected by PCR, as described (*11*).

After receiving ethics approval from the Centre Hospitalier de l’Université de Montréal–Hôpital Saint-Luc, we reviewed hospital charts and public health investigation files of patients who were harboring *Shigella* spp. with decreased susceptibility to azithromycin. Differences were analyzed by using the Fisher exact 2-tailed test with Epi Info software, version 6.0 (Centers for Disease Control and Prevention, Atlanta, GA, USA). Statistical significance was set at p<0.05.

From January 1, 2011, through April 30, 2013, a total of 45 patients were infected by 46 *Shigella* spp. strains isolated from fecal samples, including 2 also isolated from blood. A total of 33 *Shigella* spp. isolates were acquired locally by 33 men, and 13 *Shigella* spp. isolates were acquired abroad, outside Canada, in the week before symptom onset, by 6 men and 7 women (p = 0.00003).

From January 2012 through April 2013, infection with 4 *Shigella* spp. pulse types with decreased azithromycin susceptibility was locally acquired by 9 patients (mean age 45 years, range 29–55 years) (Tables 1, 2). Among these patients, 1 HIV-positive man was infected successively with 2 *Shigella* species with reduced azithromycin susceptibility, 11 months apart, resulting in a total of 10 infections (Figure). All 9 men reported having had sex with men, and 7 were HIV positive. CD4 cell counts were 320 ×10^6^ cells/L for 1 HIV-positive patient and 420–540 × 10^6^ cells/L for the other 6. HIV viral load was <40 copies/mL for 3 of the 6 patients for whom data were available and 58–90,074 copies/mL for the other 3. During the previous 6 years, 7 men for whom these data were available had experienced 1–7 (median 4) other sexually transmitted diseases. Of the 9 men, 4 reported use of sex venues and none had worked in daycare centers or as a food handler. All 9 patients received follow-up care at medical clinics outside the hospital, but 4 patients received care at the emergency room for 24–48 hours. For treatment, 4 patients received ciprofloxacin and 2 received azithromycin; antimicrobial drug treatment is unknown for the other 3 patients. For these 9 men, information was unknown with regard to receipt of azithromycin before illness onset, clinical outcome data, and antimicrobial drug treatment failure. Among the *Shigella* pulse types with reduced susceptibility to azithromycin, 2 originated from outbreaks among MSM (Figure), which are being investigated by Quebec public health departments and LSPQ.

**Table 1:** Azithromycin susceptibility of 26 *Shigella* spp. isolates from 25 patients, Centre Hospitalier de l’Université de Montréal– Hôpital Saint-Luc, Montreal, Quebec, Canada, January 2012–April 2013*

Azithromycin susceptibility	No. infections acquired locally	No. infections acquired abroad
Reduced	10	0
Susceptible	7	9

*p = 0.0039. All patients with locally-acquired *Shigella* and 3 patients with *Shigella* infections acquired abroad were men. One patient was infected successively with 2 *Shigella* species with reduced azithromycin susceptibility. For all patients infected with *Shigella* spp. with decreased azithromycin susceptibility, the strains were isolated from feces and none from blood.

**Table 2:** Characteristics and antimicrobial susceptibility of 4 *Shigella* isolates with reduced azithromycin susceptibility, Montreal, Quebec, Canada, January 2012–April 2013*

*Shigella* species	ST	PV†	AZM, mg/L‡	AMP, mg/L	TMP/SMX, mg/L	CIP mg/L	CRO, mg/L	TET, mg/L	CHL, mg/L	NAL mm
*S. flexneri*	2a	15	256	<2 (S)	>320 (R)	0.016	<1	>128	0.5 (S)	27
*S. flexneri*	2a	16	64	>32 (R)	>320 (R)	0.016	<1	>128	128 (R)	27
*S. flexneri*	3a	6	>256	>32 (R)	<20 (S)	0.016	<1	>128	>256 (R)	24–28
*S. sonnei*	–	101,105§	>256	>32 (R)	>320 (R)	0.016	<1	>128	>256 (R)	23–27

*The susceptibility and resistance break points for AMP, CIP, TMP/SMX, CRO, TET, CHL, and NAL were Clinical and Laboratory Standards Institute *Enterobacteriaceae* break points (*12*). ST, serotype; PV, pulsovar; AZM, azithromycin; AMP, ampicillin; TMP/SMX, trimethoprim/sulfamethoxazole; CIP, ciprofloxacin; CRO, ceftriaxone; TET, tetracycline; CHL, chloramphenicol; NAL, nalidixic acid; S, susceptible; R: resistant; –, not applicable. †PV was determined by *Xba*I and *Bln*I pulsed-field gel electrophoresis patterns. ‡The criterion for elevated azithro MIC was >16 mg/L (*13*). §*S. sonnei* PVs 101 and 105 were related and had 2 different pulsed-field gel electrophoresis bands.

**Figure:**
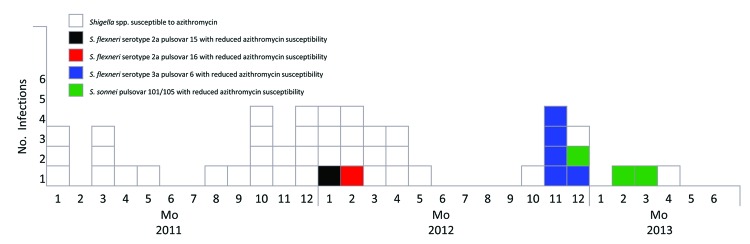
Distribution of *Shigella* spp. infections by sample date and years, Montreal, Quebec, Canada, January 2011–April 2013.

During the 2011–2013 surveillance period, azithromycin MICs for 35 of 36 *Shigella* spp. isolates with no reduced azithromycin susceptibility were 2–8 mg/L, and the MIC for 1 isolate was 16 mg/L; this latter isolate was negative by PCR for *mph*(A), and the other 35 isolates were not tested. The 10 *Shigella* spp. isolates with reduced azithromycin susceptibility had azithromycin MICs ≥64 mg/L and were positive for the *mph*(A) gene by PCR. The 3 *S. flexneri* and 1 *S. sonnei* pulse types were susceptible to nalidixic acid, ciprofloxacin, and ceftriaxone (Table 2); 3 pulse types were resistant to ampicillin, trimethoprim/sulfamethoxazole, or chloramphenicol; and 4 pulse types were resistant to tetracycline (Table 2). During 2012–2013, *Shigella* spp. with reduced azithromycin susceptibility represented 57.1% of 7 locally acquired pulse types (data not shown). Pulse-Net Canada *Xba*I and *Bln*I pattern designations were SFXXAI.0205/SFXBNI.0092 and SFXXAI.0204/SFXBNI.0093 for *S. flexneri* serotype 2a pulsovars 15 and 16, respectively; SFXXAI.0193/SFXBNI.0084 for *S. flexneri* serotype 3a pulsovar 6; SSOXAI.0395/SS0BNI.0020 for *S. sonnei* pulsovar 101; and SSOXAI.0174/SSOBNI.0176 for *S. sonnei* pulsovar 105. No PFGE matches were identified in isolates from other Canada provinces.

## Conclusions

During 2012–2013, at the Centre Hospitalier de l’Université de Montréal–Hôpital Saint-Luc, 10 infections with 1 of the 4 *Shigella* spp. pulse types with reduced azithromycin susceptibility were documented for 9 MSM, 7 of whom were HIV positive. These 4 locally acquired *Shigella* pulse types had increased azithromycin MICs of ≥64 mg/L and were positive by PCR for *mph*(A). This gene, which encodes macrolide-inactivating 2′-phosphotransferase, occurs on various plasmids (*7*). It has been documented in many aerobic gram-negative rods, such as *Escherichia coli* and *Shigella* spp. (*14*). This gene was harbored by all *Shigella* spp. with azithromycin MICs >16 mg/L (*7*,*13*–*15*). Azithromycin treatment failure has been reported for patients who received this drug for infection with such isolates (*14*). In our study, the acquisition of this gene by >50% of locally acquired *Shigella* spp. pulse types, infecting MSM over 15 months, is a concern in view of the potentially rapid development of reduced *Shigella* spp. susceptibility to azithromycin. For facilitation of clinical decision making and surveillance, azithromycin susceptibility break points for *Enterobacteriaceae* should be standardized (*12*). MSM should be counseled about prevention of enteric sexually transmitted infections; prevention measures include handwashing and using barriers during oral, anal, and genital sex (*2*,*3*). Such counseling might lead to behavior changes that might help slow the transmission of enteric sexually transmitted infections, including *Shigella* spp. infections with reduced azithromycin susceptibility. 

## References

[1] DuPont HL. *Shigella* species (bacillary dysentery). In: Mandell GL, Bennett JE, Dolin R, editors. Principles and practice of infectious diseases, 7th ed. Philadelphia: Elsevier Churchill Livingstone; 2010. p. 2905–10.

[2] Gaudreau C, Bruneau A, Ismaïl J. Outbreak of *Shigella flexneri* and *Shigella sonnei* enterocolitis in men who have sex with men, Québec, 1999 to 2001. Can Commun Dis Rep. 2005;31:85–90 . 15875326

[3] Gaudreau C, Ratnayake R, Pilon PA, Gagnon S, Roger M, Lévesque S. Ciprofloxacin-resistant *Shigella sonnei* among men who have sex with men, Canada, 2010. Emerg Infect Dis. 2011;17:1747–50 . 10.3201/eid1709.102034 21888811 PMC3322076

[4] American Academy of Pediatrics. *Shigella* infections. In: Pickering LK, Baker CJ, Kimberlin DW, Long SS, editors. Red book, 2012 Report of the Committee on Infectious Diseases, 29th ed. Elk Grove Village (IL): The Academy; 2012. p. 645–7.

[5] World Health Organization. Guidelines for the control of shigellosis, including epidemics due to *Shigella dysenteriae* type 1. Geneva: The Organization; 2005.

[6] Khan WA, Seas C, Dhar U, Salam MA, Bennish ML. Treatment of shigellosis: V. Comparison of azithromycin and ciprofloxacin. Ann Intern Med. 1997;126:697–703. 10.7326/0003-4819-126-9-199705010-00004 9139555

[7] Howie RL, Folster JP, Bowen A, Barzilay EJ, Whichard JM. Reduced azithromycin susceptibility in *Shigella sonnei*, United States. Microb Drug Resist. 2010;16:245–8. 10.1089/mdr.2010.0028 20624094

[8] Nataro JP, Bopp CA, Fields PI, Kaper JB, Strockbine NA. *Escherichia, Shigella,* and *Salmonella.* In: Versalovic J, Carroll KC, Funke G, Jorgensen JH, Landry ML, Warnock DW, editors. Manual of clinical microbiology, 10th ed. Washington (DC): American Society for Microbiology; 2011. p. 603–26.

[9] Ewing WH. The genus *Shigella*. In: Ewing W, Edwards PR, editors. Edwards and Ewing’s identification of *Enterobacteriaceae,* 4th ed. New York: Elsevier Scientific Publishing Co., Inc.; 1986. p. 135–72.

[10] Ribot EM, Fair MA, Gautom R, Cameron DN, Hunter SB, Swaminathan B, Standardization of pulsed-field gel electrophoresis protocols for the subtyping of *Escherichia coli* O157:H7, *Salmonella* and *Shigella* for PulseNet. Foodborne Pathog Dis. 2006;3:59–67. 10.1089/fpd.2006.3.59 16602980

[11] Ojo KK, Ulep C, Van Kirk N, Luis H, Bernardo M, Leitao J, The *mef*(A) gene predominates among seven macrolide resistance genes identified in gram-negative strains representing 13 genera, isolated from healthy Portuguese children. Antimicrob Agents Chemother. 2004;48:3451–6. 10.1128/AAC.48.9.3451-3456.2004 15328110 PMC514787

[12] Clinical and Laboratory Standards Institute. Performance standards for antimicrobial susceptibility testing; 23th informational suppplement; no. M100–S23, vol. 33, no. 1. Wayne (PA): The Institute; 2013.

[13] Sjölund Karlsson M, Bowen A, Reporter R, Folster JP, Grass JE, Howie RL, Outbreak of infections caused by *Shigella sonnei* with reduced susceptibility to azithromycin in the United States. Antimicrob Agents Chemother. 2013;57:1559–60. 10.1128/AAC.02360-12 23274665 PMC3591876

[14] Boumghar-Bourtchai L, Mariani-Kurkdjian P, Bingen E, Filliol I, Dhalluin A, Ifrane SA, Macrolide-resistant *Shigella sonnei.* Emerg Infect Dis. 2008;14:1297–9. 10.3201/eid1408.080147 18680661 PMC2600399

[15] Centers for Disease Control and Prevention. Outbreak of infections caused by *Shigella sonnei* with decreased susceptibility to azithromycin—Los Angeles, California, 2012. MMWR Morb Mortal Wkly Rep. 2013;62:171 . 23466436 PMC4604789

